# Psychosocial consequences of screening-detected abdominal aortic aneurisms: a cross-sectional study

**DOI:** 10.1080/02813432.2021.2004713

**Published:** 2021-11-21

**Authors:** Christina Sadolin Damhus, Volkert Siersma, Anders Hansson, Christine Winther Bang, John Brodersen

**Affiliations:** a The Section and Research Unit for General Practice, Department of Public Health, University of Copenhagen, Copenhagen, Denmark; b Primary & eHealth Care, Region Zealand, Denmark; c Department of Public Health and Community Medicine, Institute of Medicine, The Sahlgrenska Academy, University of Gothenburg, Gothenburg, Sweden; d University Health Care Research Center, Faculty of Medicine and Health, Orebro University, Orebro, Sweden

**Keywords:** Screening, abdominal aortic aneurysm, psychosocial consequences, COS-AAA questionnaire, survey study

## Abstract

**Objective:**

In Sweden, an abdominal aortic aneurysm (AAA) screening programme was gradually implemented from 2009 to reduce the incidence of rupture and thereby mortality. AAA screening introduces a variety of unintended, but generally unavoidable, harms, e.g. stress and worry. Such psychosocial consequences have previously only been investigated with generic measures. Therefore, the aim of this study was to describe and compare the psychosocial consequences in men with a screening detected AAA to men with a normal screening result after they participated in the Swedish national AAA-screening programme using a validated psychometric instrument.

**Material and methods:**

This study was a cross-sectional survey. Data were originally collected to validate the COS-AAA and has previously been published in details. The Consequences of Screening in Abdominal Aortic Aneurysm (COS-AAA) questionnaire was sent to 250 men with a screening detected AAA and 500 with a normal screening result who were randomly selected from a Swedish population-based screening register.

**Results:**

In total, 158 (63%) men with a screening detected AAA and 275 (55%) men with a normal screening result completed the COS-AAA. We found that men with a screening detected AAA reported negative psychosocial consequences to a greater extent in 10 of 13 COS-AAA Part 1 scales, all statistically significant except three (behaviour, sleep and negative experiences from examination). For COS-AAA Part 2, there was a statistically significant difference between groups in four of five scales.

**Conclusions:**

Men diagnosed with a screening detected AAA, reported more negative psychosocial consequences compared to men with a normal result. Screening for abdominal aorta aneurism (AAA) introduces intended benefits and unintended harms. Adequate measures are necessary to determine the balance between them.

**Key points:**
- This study applied a condition-specific questionnaire with high content validity and adequate psychometric properties to measure psychosocial consequences in men participating in AAA screening.
- We found that men with a screening detected AAA reported more negative psychosocial consequences than men with a normal aorta size.
- The risk of negative psychosocial consequences is important to include in the decision making on whether to participate in screening or not.

## Background

Abdominal aortic aneurysms (AAAs), localized enlargements of the abdominal aorta, are often asymptomatic until they rupture, but can thereafter turn into a life-threatening condition with about 80% mortality [1]. In 85–90% of the cases, an aneurysm is caused by atherosclerosis due to smoking and the affected patients are often already diagnosed with a cardiovascular or chronic obstructive pulmonary disease [1]. The high mortality rate combined with the fact that the disease rarely gives symptoms makes screening appealing as screening aims to detect the aneurysm before it ruptures enabling preventive surgery. Therefore, the intended benefit of AAA screening is to reduce the overall death rate from rupture. Since women typically develop atherosclerosis later in life, the benefit of screening for AAA in women is lower, therefore implemented screening programmes have exclusively targeted men [1]. Population-based screening has gradually been implemented in the United States from 2005, in Sweden from 2009 and in the United Kingdom from 2013. The implementations were based on four randomised controlled trials, which found that screening men aged 65 years or older decreased AAA-related mortality rates by approximately 50% over 13–15 years [2–5]. It is undetermined whether, this screening also reduces all-cause mortality [6]. In Sweden, AAA screening includes a one-time ultrasound examination for men aged 65 years. According to the National Board of Health and Welfare in Sweden, the recommended procedure depends on the size of the aneurysm. Men with small aneurysms between 30 and 54 mm are offered continued follow-up, and men diagnosed with aneurysms with a diameter of ≥55 mm are offered elective preventive surgery. Screening has doubled the use of preventive surgery which is not without danger as it has a mortality-risk of 3.9–4.5% and an estimated overall complication rate of 32% [7].

A study reported that 84% of men accepted the invitation to the Swedish AAA screening programme in 2000–2014, and the prevalence of AAA was 1.5% among those screened [8]. After the introduction of AAA screening in Sweden, a risk reduction in AAA-related mortality of 4% per year has been reported [8]. However, Johansson and colleagues reported that AAA screening did not contribute substantially to the reduction and that it was probably caused by factors as reduced smoking and adjuvant medication for cardiovascular risk factors [9]. The benefits of a screening programme are offset by a variety of unintended, but generally unavoidable, harms. Notably, preventive surgery prevents AAA from rupture but also, it introduces the risk of perioperative complications such as myocardial infarction, stroke and reoperation for bleeding. Another harm of AAA screening is the psychosocial impact, which both qualitative and quantitative studies have investigated with mixed conclusions. In this context, we refer to screening as the whole cascade of events in the screening programme as each element has the power to impact the psychosocial consequences. The conceptualization of psychosocial consequences is based on the bio-psychosocial model in which people are not regarded as passive, but considered able to both interact with and influence the environment [10]. Qualitative studies [11,12] found that men experienced anxiety towards the risk of rupture or as a man described it ‘a ticking bomb inside your stomach’ [13]. Other reported consequences were worries, existential thoughts about life’s fragility and mortality due to the AAA diagnosis. Quantitative studies used generic questionnaires, e.g. SF-36, ScreenQL, EQ-5D and HADS to measure psychological aspects and quality of life (QoL) following AAA screening [4,14–18]. Such generic questionnaires aim to measure QoL but lack content validity in the context of measuring potential psychosocial consequences of AAA-screening, because they are not purposely targeted to measure the psychosocial aspects that are specifically important for AAA-screened men [19]. In a previous publication [20], we developed and validated the COS-AAA which is a condition-specific questionnaire to measure psychosocial consequences in men who undergo AAA screening. Psychosocial consequences are important to acknowledge and measure using a valid instrument as they have the potential to shift the benefit-harm ratio of medical screening. Therefore, the aim of this study was to describe and compare psychosocial consequences in men with a screening detected AAA, to men with a normal screening result after they participated in the Swedish national AAA screening programme, using a condition-specific questionnaire with high content validity and adequate psychometric properties for measurement of psychosocial consequences in AAA screening.

## Methods

### Setting and study participants

Data in this study were originally collected to validate the COS-AAA which has previously been published in details [20]. In short, men eligible for this study participated in the AAA screening programme of Western Sweden (County of Västra Götaland). The exclusion criteria were (1) not being able to read and understand the Swedish language, or (2) already having had a registered AAA detected before the screening. All written material was in Swedish including the invitation letter and the COS-AAA questionnaire. Therefore, eligible participants not understanding the written material were expected not to participate in the survey. From January to April 2013, an informed consent document together with the COS-AAA questionnaire and a stamped-addressed envelope was posted to the 250 men who had been diagnosed with AAA in connection with screening within a period of 1‒24 months. Furthermore, to a group of 500 men with normal aorta according to screening, who were randomly selected from a population-based screening register from the same period. We performed a consecutive sampling, meaning that every men meeting our inclusion criteria were selected until the required sample size was achieved. A reminder was sent if no response was received after about 2 weeks.

### The COS-AAA

To measure the psychosocial consequences, the COS-AAA was used (Supplementary 1). This questionnaire was specifically developed to measure psychosocial consequences of screening for AAA, has high content validity and is statistically validated using item response theory, Rasch models [20]. COS-AAA has two parts, and measures the following scales (number of items): *Part 1*, Anxiety (7), Behavioural (7), Dejection (6), Sleep (3), Uncertainty about the result of the ultrasound examination (3), Change in body perception (8), Guilt (2), Fear and powerlessness (7), Negative experiences from the examination (2), Emotional reactions (3), Change in lifestyle (2), Better not knowing (2), Fear of rupture (4), Sexuality (3), Information (2), Stigmatised (4), Self-blame (3), and Regretful still smoking (6); *Part 2*, Existential values ​​(6), Relaxed/calm (3), Social relations (3), Impulsivity (6), and Empathy (3). Part 1 can be used before, during and after screening. The items have four answer options―Not at all, a bit, quite a bit, a lot―which score from 0 to 3 respectively. Part 2 can only be used after screening and the items have five answer options―much less, less, same as before, more, much more―with scoring from 2, 1, 0, 1, and 2 respectively. These scales measure the tendency of change, and it is therefore undetermined whether this change is positive or negative.

### Covariates

Information on the following sociodemographic factors: marital status, native language, education, household income and smoking and information on self-rated health was obtained from a questionnaire sent to participants together with the COS-AAA. Marital status was grouped into single, married/cohabitating couple, couple living apart, widow. Education was measured as the highest attained education and divided into; elementary school, high school > 2 years, high school > 3 years, university < 3 years, and university > 3 years. Household income in Swedish crowns were dived into; <14 999, 15 000–29 000, 30 000–44 999, 45 000–59 999, and >60 000. Smoking into two categories: none/former smokers and current smokers. Self-rated health could be answered with the following options; excellent, very good, good, fair or poor.

### Analysis

Differences in selected characteristics of the study participants were tested between the group of men with screening detected AAA and the group of men with normal screening results by chi-squared tests with Monte Carlo estimation of the *p*-value (Table 1). Differences in the dimensions of COS-AAA between the two groups were denoted by means and standard deviations (SD) in each of the groups and were tested by non-parametric Wilcoxon signed-rank tests (Table 2). Further, differences between the means in the two groups were adjusted for covariates in a multivariable linear regression analysis (Table 2). Benjamini–Hochberg procedure was used to account for multiple testing. The scales, Uncertainty about the result of the examination (3-item), Guilt (2-item), Better not knowing (2-item), Fear of rupture (4-item) and Sexuality (3-item), are only relevant for AAA-detected men, therefore results from men with normal screening results were not reported. For the analyses SAS version 9.4 (SAS Inc., Cary, NC) was used.

**Table 1.** Characteristics of the study participants, men with AAA and men with normal screening result.

Characteristics	AAA (*n* = 158)	Normal (*n* = 275)	*p*-value
Marital status (%)			
Single	12.2	11	0.0781
Married/cohabitating couple	81.4	80.6	
Couple living apart	1.3	5.5	
Widow	5.1	2.9	
Native language (%)			
Swedish	92.4	93.8	0.5593
Other	7.6	6.2	
Education (%)			
Elementary school	51.1	46.7	0.0226
High schoo*l* > 2 years	10.9	8.1	
High schoo*l* > 3 years	14.1	15.4	
Universit*y* < 3 years	10.3	11	
Universit*y* > 3 years	7.7	18.8	
Household income in (SEK) (%)			
< 14,999	13.2	6.3	0.0298
15,000–29,000	35.1	29.0	
30,000–44,999	25.8	27.2	
45,000–59,999	16.6	22.8	
60,000 >	9.3	14.7	
Smoking (%)			
None/former smokers	67	71	0.3072
Current smokers	33	29	
Self-rated health (%)			
Excellent	7.1	15.4	0.0000
Very good	20.5	34.9	
Good	42.9	38.6	
Fair	25.0	8.5	
Poor	4.5	2.6	

Percentages and *p*-value.

**Table 2.** Part 1 and Part 2.

Scales		AAA		Normal		*p*-value	Adjusted^a^	*p*-value
*Part 1. COS-AAA*								
Scales	Range	Mean	SD	Mean	SD	Sign.	Mean diff (95%CI)	Sign.
Anxiety (7-item)	0–21	1.86	2.61	0.96	1.44	<0.001*	0.46 (0.11 ; 0.81)	0.0093*
Sense of dejection (6-item)	0–18	1.99	2.66	1.11	1.73	<0.001*	0.44 (0.05 ; 0.83)	0.0280*
Behaviour (7-item)	0–21	2.25	2.92	1.22	1.87	<0.001*	0.40 (−0.02 ; 0.82)	0.0624
Sleep (3-item)	0–9	1.59	1.84	1.29	1.63	0.0909	0.13 (−0.21 ; 0.47)	0.4507
Uncertainty about the result of the examination (3-item)	0–9	1.78	1.46					
Change in body perception (8-item)	0–24	3.29	3.01	1.63	2.32	<0.001*	1.05 (0.60 ; 1.49)	<0.001*
Guilt (2-item)	0–6	0.55	0.88					
Fear and powerlessness (7-item)	0–21	2.21	2.89	0.79	1.82	<0.001*	0.80 (0.43 ; 1.16)	<0.001*
Negative experiences from the examination (2-item)	0–6	0.21	0.54	0.11	0.56	0.0590	0.02 (−0.08 ; 0.12)	0.7083
Emotional reactions (3-item)	0–9	1.06	1.44	0.59	0.99	<0.001*	0.26 (0.03 ;0.50)	0.0248*
Change in lifestyle (2-item)	0–6	0.79	1.04	0.48	0.89	<0.001*	0.25 (0.06 ; 0.44)	0.0108*
Better not knowing (2-item)	0–6	0.18	0.67					
Fear of rupture (4-item)	0–12	1.66	1.92					
Sexuality (3-item)	0–9	1.40	2.11					
Information (2-item)	0–6	0.61	1.14	0.04	0.28	<0.001*	0.61 (0.45 ; 0.76)	<0.001*
Stigmatised (4-item)	0–12	3.44	5.57	4.77	7.17	0.0324*	−1.61 (−3.02 ; −0.20)	0.0256*
Self-blame (3-item)	0–9	2.99	4.23	3.64	5.33	0.1693	−0.91 (−1.98 ; 0.15)	0.0932
Regretful (6-item)	0–18	2.46	2.16	4.21	2.32	<0.001*	−1.54 (−2.56 ; −0.52)	0.0031*
*Part 2. COS-AAA*								
Existential values (6-item)	0–12	1.23	2.03	0.48	1.36	<0.001*	0.63 (0.31 ; 0.95)	<0.001*
Relaxed/calm (2-item)	0–4	0.52	0.93	0.32	0.69	0.0262*	0.18 (0.01 ; 0.34)	0.0332*
Social relations (3-item)	0–6	0.12	0.59	0.04	0.27	0.0861	0.09 (0.00 ; 0.17)	0.0465
Impulsivity (6-item)	0–12	0.86	1.85	0.18	0.82	<0.001*	0.62 (0.37 ; 0.87)	<0.001*
Empathy (3-item)	0–6	0.68	1.02	0.28	0.65	<0.001*	0.39 (0.23 ; 0.56)	<0.001*

COS-AAA, men with AAA and men with normal screening result.

^a^Adjusted in a multivariable linear regression for; marital status, native language, education, household income, smoking, self-rated health.

*Adjustment for multiple testing with the method of Benjamini–Hochberg rejects all *p*-values less than 0.0375 to control the false discovery rate at 0.05.

## Ethical considerations

The project was approved by the Regional Ethical Review Board in Gothenburg (ref. no. 403-09). All potential participants received information about the purpose of the study in writing. Participants were informed about their right to decline. Informed consent was obtained from all men before participation.

## Results

One hundred and fifty-eight men with AAA (63% of eligible) and 275 with a normal screening result (55% of eligible) completed the COS-AAA questionnaire.

There was a statistically significant difference between the groups regarding education and income, more men diagnosed with AAA had lower education and low income. There was a statistically significant difference between the groups regarding self-rated health, as the men diagnosed with AAA estimated their health lower than those with normal screening result (Table 1). No other significant differences were found (Table 1).

In the adjusted analysis, we found that men with a screening-detected AAA reported negative psychosocial consequences to a greater extent in 10 of 13 COS-AAA Part 1 scales, all statistically significant except three: Behaviour, Sleep and Negative experiences from examination (Table 2). Regarding Stigmatisation, Self-blame and Regretful the group with a normal screening result had a higher score than the AAA group, Stigmatisation and Regretful being statistically significant. For COS-AAA Part 2, there was a statistically significant difference between the AAA-group and the men with a normal screening result in four of five scales: Empathy, Impulsivity, Relaxed/calm, Existential values. Social relations being insignificant (Table 2).

## Discussion

This study used the condition-specific questionnaire COS-AAA to measure psychosocial consequences in AAA screening. We found that men with an AAA-diagnosis reported more negative psychosocial consequences in 10 of the 13 scales in Part 1, and a statistically significant difference was seen between groups in four of five scales in Part 2 of the COS-AAA.

### Strengths and limitations

A strength of this study was the use of COS-AAA: a condition-specific questionnaire with high content validity and adequate psychometric properties. There was an acceptable response rate in both groups indicating that COS-AAA was regarded as a relevant questionnaire for completion by the target group; men participating in AAA screening. This study had several limitations. First, the participants had to be able to read and understand the Swedish language, which means that we cannot say anything about potential cultural differences. Second, due to the cross-sectional design, we were not able to conclude on trends over time as our results reflect one time-point. Therefore, our study is not designed to give robust evidence about the degree and for how long potential negative psychosocial consequences will last. Ideally, psychosocial consequences are measured before the invitation to screening, after invitation but before potential participating, and after potential participating preferable at more time points e.g. at 3, 6 and 12 months after screening [21]. This has been exemplified in colorectal [22], breast [23] and lung cancer screening [24]. Third, men responded to the COS-AAA 1–24 months after screening, which might had an impact on their responses, as some might be more affected in the time just after a potential detected AAA or just before a preventive surgery. Also, aorta-size might be an important factor consider in future studies. Unfortunately, we did not have access to this information.

### Interpretation of results and comparison with other studies

Table 2 shows the results of the adjusted mean difference in COS-AAA score between the two groups. A mean difference of 1.05 points (<0.001), as seen for the scale *Change in body perception,* means that 5% of the AAA-group scored two answering categories higher than the men with a normal screening result on one of the items in the scale, and the remaining 95% of the AAA-group scored one answering category higher than the men with a normal screening result on one of the items in the scale. The AAA-group answering *‘a bit’* where the men with a normal screening result answered ‘*Not at all’,* exemplifies a hypothetical situation where the AAA-group scored one answering category higher on the item; ‘*are you worried?’* It is undetermined if these differences are of clinical importance as no established threshold exists.

It is difficult to compare previous quantitative studies to the present study, as they have used generic questionnaires to measure outcomes of AAA screening [4,14–18]. Therefore, these studies do not adequately investigate all potential psychosocial consequences of AAA screening. Five [4,14–16,18] of these studies indicated no clinically important decrease in QoL and one concluded more specifically that screening older men for AAA is not harmful to their self-perceived general health [16]. Such conclusions must be interpreted with extreme caution. First, as mentioned, the generic questionnaires lack content validity in the specific setting of AAA screening why they might not measure what is important. Second, generic questionnaires are not very sensitive because they ask into a broad range of health aspects and will have difficulties finding any differences. Third, screening involves an examination of healthy people; thus, people who do not have symptoms of the disease they are being screened for. Therefore, we argue that even a small difference in psychosocial consequences is of importance as participants have not actively sought treatment but are, apparently healthy citizens, at risk of experiencing potential harm.

In qualitative studies, Bertero et al. found that men appreciated that their aneurysm was detected and were confident and secure about being under superintendence [11]. Hansson et al. found that the men, on the one hand, appreciated the detection of hidden risk-conditions and that the information they received gave them comfort, but, on the other hand, the detection created worry, uncertainty and feelings of anxiety [13]. Pettersson’s found that patients searched for answers about how to influence the growth of the aneurysm in their everyday life [12]. Also, that the healthcare system sometimes failed to clarify follow-up routines in a way that made men with a screening-detected AAA feel safe and secure [12]. Compared to our findings, these studies indicate how screening introduce new thoughts and concerns about health and disease, some of which have negative psychosocial consequences.

Results from Part 2 in our study revealed that in four of five scales, there were statistically significant differences between the AAA-group and the group of men with a normal screening result. As these scales measure the tendency of change, it is not obvious whether these changes are positive or negative. Similar, to our results, a survey-study found that the scale ‘Existential values’ remained significantly different between women with false positives and women with normal screening results at 36-month follow-up of mammography screening [23]. Gram et al. also reported an overall positive impact on life as a long-term consequence of false-positive screening mammography [25]. Participants in this study stated that they were grateful for the experience of screening because they found life more precious afterwards. Still, the authors argued that this should not constitute a benefit of cancer screening, ‘*since first the fear, then the relief, are induced by the same screening*’ [25]. Based on this and own results, we argue that a change in psychosocial consequences among apparently healthy individuals is a negative feature of screening.

Interestingly, we found that men in the AAA group to a higher extend succeed in life style changes. This might be interpreted as a positive outcome of screening. However, the motivation to life style changes could also be interpreted as being based on FUD: fear, uncertainty and doubt [26].

### Implications for research, policy and practice

This study is important to public health as it shows that men with a diagnosis of a screened-detected AAA experienced significantly more negative psychosocial consequences than men with a normal screening result. Negative psychosocial consequences might increase the number of consultations in general practice and is thereby tiring for both the individual and the health care system. This study is important, as it draws attention to these relevant issues in screening and calls for more research using adequate measures and appropriate designs [27].

Also, a recent study found that AAA mortality decreased by more than 70% from 2005 to 2015, and this change was similar in a screened and non-screened population [9]. Further, a lower absolute number of overdiagnosed cases (49 versus 176 per 10 000 invited men) and fewer overtreated cases (19 versus 37 per 10 000 invited men) were observed compared with results of the largest earlier trial of screening for AAA (the MASS trial) [4,9]. However, since the harms of screening decreased less than the benefits, the balance between benefits and harms seem much less appealing in today’s setting [9].

Importantly, screening participants are continuously at risk of potential psychosocial consequences, even when the benefit of screening is reduced. Only future longitudinal designs can estimate the magnitude and duration of these psychosocial consequences. Results from these studies, combined with the decreased benefits of the screening programme, should be included when policy makers responsible for the screening guidelines evaluate the balance between benefits and harms of the AAA screening programme.

The results on psychosocial consequences are relevant when informing eligible participants about the screening programme. Psychosocial consequences are often not mentioned when inviting citizens to screening programmes but should be included to support an informed decision about screening. It is essential to include both the benefits and harms in such material as research shows that participants tend to downplay the harms of screening [28,29].

## Conclusions

This study found that men with a screening-detected AAA reported psychosocial consequences to a greater extent compared with men having a normal screening result. This trend was recognised in 10 of 13 scales in Part 1, COS-AAA, all statistically significant except three scales. For Part 2, COS-AAA, we found a statistically significant difference between groups in four of five scales which we argue can be interpreted as a negative change. These results call for more research using adequate measures and appropriate designs to unfold and estimate the magnitude and duration of psychosocial consequences in AAA screening programmes.

## Supplementary Material

Supplemental Material

## Data Availability

Please contact the corresponding author for data requests.

## References

[1] Reimerink JJ, van der Laan MJ, Koelemay MJ, et al. Systematic review and meta-analysis of population-based mortality from ruptured abdominal aortic aneurysm. Br J Surg. 2013;100(11):1405–1413. 24037558 10.1002/bjs.9235

[2] Norman PE, Jamrozik K, Lawrence-Brown MM, et al. Population based randomised controlled trial on impact of screening on mortality from abdominal aortic aneurysm. BMJ. 2004;329(7477):1259. 15545293 10.1136/bmj.38272.478438.55 PMC534438

[3] Lindholt JS, Sørensen J, Søgaard R, et al. Long-term benefit and cost-effectiveness analysis of screening for abdominal aortic aneurysms from a randomized controlled trial. Br J Surg. 2010;97(6):826–834. 20473995 10.1002/bjs.7001

[4] Ashton HA, Buxton MJ, Day NE, Multicentre Aneurysm Screening Study Group, et al. The Multicentre Aneurysm Screening Study (MASS) into the effect of abdominal aortic aneurysm screening on mortality in men: a randomised controlled trial. Lancet. 2002;360(9345):1531–1539. 12443589 10.1016/s0140-6736(02)11522-4

[5] Thompson SG, Ashton HA, Gao L, Multicentre Aneurysm Screening Study (MASS) Group, et al. Final follow-up of the Multicentre Aneurysm Screening Study (MASS) randomized trial of abdominal aortic aneurysm screening. Br J Surg. 2012;99(12):1649–1656. 23034729 10.1002/bjs.8897 PMC3569614

[6] Lederle FA. Does abdominal aortic aneurysm screening save lives? JAMA Surg. 2016;151(8):697–698. 27119312 10.1001/jamasurg.2016.0044

[7] Guirguis-Blake JM, Beil TL, Senger CA, et al. Ultrasonography screening for abdominal aortic aneurysms: a systematic evidence review for the U.S. Preventive services task force. Ann Intern Med. 2014;160(5):321–329. 24473919 10.7326/M13-1844

[8] Wanhainen A, Hultgren R, Linne A, On behalf of the Swedish Aneurysm Screening Study Group (SASS), et al. Outcome of the Swedish Nationwide Abdominal Aortic Aneurysm Screening Program. Circ. 2016;134(16):1141–1148. 10.1161/CIRCULATIONAHA.116.022305 27630132

[9] Johansson M, Zahl PH, Siersma V, et al. Benefits and harms of screening men for abdominal aortic aneurysm in Sweden: a registry-based cohort study. Lancet. 2018;391(10138):2441–2447. 29916384 10.1016/S0140-6736(18)31031-6

[10] Brodersen J, Thorsen H. Consequences of Screening in Breast Cancer (COS-BC): development of a questionnaire. Scand J Prim Health Care. 2008;26(4):251–256. 19034808 10.1080/02813430802542508 PMC3406644

[11] Bertero C, Carlsson P, Lundgren F. Screening for abdominal aortic aneurysm, a one-year follow up: an interview study. J Vasc Nurs. 2010;28(3):97–101. 20709266 10.1016/j.jvn.2010.04.001

[12] Pettersson M, Hansson A, Brodersen J, et al. Experiences of the screening process and the diagnosis abdominal aortic aneurysm among 65-year-old men from invitation to a 1-year surveillance. J Vasc Nurs. 2017;35(2):70–77. 28527730 10.1016/j.jvn.2016.11.003

[13] Hansson A, Brodersen J, Reventlow S, et al. Opening Pandora's box: the experiences of having an asymptomatic aortic aneurysm under surveillance. Health Risk Soc. 2012;14(4):341–359.

[14] Lucarotti M, Heather B, Shaw E, et al. Psychological morbidity associated with abdominal aortic aneurysm screening. Eur J Vasc Endovasc Surg. 1997;14(6):499–501. 9467527 10.1016/s1078-5884(97)80131-1

[15] Lindholt JS, Vammen S, Fasting H, et al. Psychological consequences of screening for abdominal aortic aneurysm and conservative treatment of small abdominal aortic aneurysms. Eur J Vasc Endovasc Surg. 2000;20(1):79–83. 10906303 10.1053/ejvs.1999.1087

[16] Spencer CA, Norman PE, Jamrozik K, et al. Is screening for abdominal aortic aneurysm bad for your health and well-being? ANZ J Surg. 2004;74(12):1069–1075. 15574151 10.1111/j.1445-1433.2004.03270.x

[17] Wanhainen A, Rosén C, Rutegård J, et al. Low quality of life prior to screening for abdominal aortic aneurysm: a possible risk factor for negative mental effects. Ann Vasc Surg. 2004;18(3):287–293. 15354629 10.1007/s10016-004-0021-x

[18] Lesjak M, Boreland F, Lyle D, et al. Screening for abdominal aortic aneurysm: does it affect men's quality of life? Aust J Prim Health. 2012;18(4):284–288. 22951209 10.1071/PY11131

[19] Brodersen J, Doward L, Thorsen H, et al. Patient reported outcome scales for health sciences: from medical paternalism to patient-autonomy. Rasch Relat Models Methods Health Sci. 2009;5:281–302.

[20] Brodersen J, Hansson A, Johansson M, et al. Consequences of screening in abdominal aortic aneurysm: development and dimensionality of a questionnaire. J Patient Rep Outcomes. 2017;2:37. 30238082 10.1186/s41687-018-0066-1 PMC6120857

[21] DeFrank JT, Barclay C, Sheridan S, et al. The psychological harms of screening: the evidence we have versus the evidence we need. J Gen Intern Med. 2015;30(2):242–248. 25150033 10.1007/s11606-014-2996-5 PMC4314481

[22] Malmquist J. Psychosocial consequences of colorectal cancer screening: adequacy of measurement and effects. Copenhagen: University of Copenhagen; 2020.

[23] Brodersen J, Siersma VD. Long-term psychosocial consequences of false-positive screening mammography. Ann Fam Med. 2013;11(2):106–115. 23508596 10.1370/afm.1466 PMC3601385

[24] Rasmussen JF, Siersma V, Pedersen JH, et al. Psychosocial consequences in the Danish randomised controlled lung cancer screening trial (DLCST). Lung Cancer. 2015;87(1):65–72. 25433982 10.1016/j.lungcan.2014.11.003

[25] Gram I, Lund E, Slenker S. Quality of life following a false positive mammogram. Br J Cancer. 1990;62(6):1018–1022. 2257206 10.1038/bjc.1990.430 PMC1971570

[26] Østerø J, Siersma V, Brodersen J. Breast cancer screening implementation and reassurance. Eur J Public Health. 2014;24(2):258–263. 23788014 10.1093/eurpub/ckt074

[27] Cotter AR, Vuong K, Mustelin LL, et al. Do psychological harms result from being labelled with an unexpected diagnosis of abdominal aortic aneurysm or prostate cancer through screening? A systematic review. BMJ Open. 2017;7(12):e017565. 10.1136/bmjopen-2017-017565 PMC5728272 29237653

[28] Byskov Petersen G, Sadolin Damhus C, Ryborg Jønsson AB, et al. The perception gap: how the benefits and harms of cervical cancer screening are understood in information material focusing on informed choice. Health Risk Soc. 2020;22(2):177–196.

[29] Damhus CS, Byskov Petersen G, Ploug T, et al. Informed or misinformed choice? Framing effects in a national information pamphlet on colorectal cancer screening. Health Risk Soc. 2018;20(5–6):241–258.

[30] World Medical Association. World Medical Association Declaration of Helsinki. Ethical principles for medical research involving human subjects. Bull World Health Org. 2001;79(4):373–374. 11357217 PMC2566407

