# Supplementary material for: Psychosocial consequences of screening-detected abdominal aortic aneurisms: a cross-sectional study
Source: Scand J Prim Health Care. 2021 Nov 21;39(4):459–65. doi: 10.1080/02813432.2021.2004713 (PMC8725974; doi:10.1080/02813432.2021.2004713)
Supplement: Supplemental Material [file IPRI_A_2004713_SM6320.docx]

**Supplementary 1**

**Consequences of screening – Abdominal aorta aneurysm (COS-AAA)**

The following is an ad hoc translation of the COS-AAA Part 1+2. Regarding psychometric standards, the validity of the English translation is not inherited from the Swedish original questionnaire. To be validated, the translation must follow certain methods and the resulting translated questionnaire has to be validated in a relevant English-speaking group.

Copyright of the Swedish version of the COS-AAA questionnaire belongs to Anders Hansson, Monica Pettersson and John Brodersen.

**COS AAA, Part 1**

| Have you, **during the last week**, experienced any of the following? | Not  at all | A bit | Quite a bit | A lot |
| --- | --- | --- | --- | --- |
| 1. I have been worried. | 🞏 | 🞏 | 🞏 | 🞏 |
| 2. I have been worried about my future. | 🞏 | 🞏 | 🞏 | 🞏 |
| 3. I have been scared. | 🞏 | 🞏 | 🞏 | 🞏 |
| 4. I have been irritable. | 🞏 | 🞏 | 🞏 | 🞏 |
| 5. I have been quieter than normal. | 🞏 | 🞏 | 🞏 | 🞏 |
| 6. I have slept badly. | 🞏 | 🞏 | 🞏 | 🞏 |
| Have you, **during the last week**, experienced any of the following? | Not  at all | A bit | Quite a bit | A lot |
| 7. I have had difficulties concentrating. | 🞏 | 🞏 | 🞏 | 🞏 |
| 8. I have felt that time passed slowly. | 🞏 | 🞏 | 🞏 | 🞏 |
| 9. My appetite has changed. | 🞏 | 🞏 | 🞏 | 🞏 |
| 10. I have been sad. | 🞏 | 🞏 | 🞏 | 🞏 |
| 11. I have been upset. | 🞏 | 🞏 | 🞏 | 🞏 |
| 12. I have been restless. | 🞏 | 🞏 | 🞏 | 🞏 |
| Have you, **during the last week**, experienced any of the following? | Not  at all | A bit | Quite a bit | A lot |
| 13. I have been nervous. | 🞏 | 🞏 | 🞏 | 🞏 |
| 14. I have been uneasy. | 🞏 | 🞏 | 🞏 | 🞏 |
| 15. It has taken a long time to fall asleep. | 🞏 | 🞏 | 🞏 | 🞏 |
| 16. I have withdrawn into myself. | 🞏 | 🞏 | 🞏 | 🞏 |
| 17. I have been unable to cope. | 🞏 | 🞏 | 🞏 | 🞏 |
| 18. I have been depressed. | 🞏 | 🞏 | 🞏 | 🞏 |

| Have you, **during the last week**, experienced any of the following? | Not  at all | A few times | Some-times | Many times |
| --- | --- | --- | --- | --- |
| 19. I have tried to stay busy to take my mind of things. | 🞏 | 🞏 | 🞏 | 🞏 |
| 20. I have had difficulties dealing with work or other commitments. | 🞏 | 🞏 | 🞏 | 🞏 |
| 21. I have had difficulties doing things around the  House. | 🞏 | 🞏 | 🞏 | 🞏 |
| 22. I have been terrified. | 🞏 | 🞏 | 🞏 | 🞏 |
| 23. I have woken up far too early in the morning. | 🞏 | 🞏 | 🞏 | 🞏 |
| 24. I have been awake most of the night. | 🞏 | 🞏 | 🞏 | 🞏 |
| Have you, **during the last week**, experienced any of the following? | Not  at all | A bit | Quite  a bit | A lot |
| 25. I have been angry. | 🞏 | 🞏 | 🞏 | 🞏 |
| 26. I have felt frail. | 🞏 | 🞏 | 🞏 | 🞏 |
| 27. After ultrasound I regret that I was examined. | 🞏 | 🞏 | 🞏 | 🞏 |
| 28. I have been sour. | 🞏 | 🞏 | 🞏 | 🞏 |
| 29. I think the examination has been uncomfortable. | 🞏 | 🞏 | 🞏 | 🞏 |
| Have you, **during the last week**, experienced any of the following? | Not  at all | A bit | Quite  a bit | A lot |
| 30. I have thought my body was vulnerable. | 🞏 | 🞏 | 🞏 | 🞏 |
| 31. I have paying attention to whether my body feels different. | 🞏 | 🞏 | 🞏 | 🞏 |
| 32. It came as a surprise to me that something was wrong. | 🞏 | 🞏 | 🞏 | 🞏 |
| 33. It feels like it is my own fault. | 🞏 | 🞏 | 🞏 | 🞏 |
| 34. The fear that the aneurism will rupture has been in the back of my mind. | 🞏 | 🞏 | 🞏 | 🞏 |
| 35. I have been worried that the aneurysm will rupture at hard physical activities. | 🞏 | 🞏 | 🞏 | 🞏 |
| 36. I have changed exercise habits. | 🞏 | 🞏 | 🞏 | 🞏 |
| 37. I have felt insecure. | 🞏 | 🞏 | 🞏 | 🞏 |

| Have you, **during the last week**, experienced any of the following? | Not  at all | | A bit | | Quite  a bit | | A lot | |
| --- | --- | --- | --- | --- | --- | --- | --- | --- |
| 38. I have thought my body was vulnerable. | 🞏 | | 🞏 | | 🞏 | | 🞏 | |
| 39. I have been uncertain about what the results of the ultrasound examination really means. | 🞏 | | 🞏 | | 🞏 | | 🞏 | |
| 40. I have had difficulties in accepting that the examination result was correct. | 🞏 | | 🞏 | | 🞏 | | 🞏 | |
| 41. I have thought about death. | 🞏 | | 🞏 | | 🞏 | | 🞏 | |
| Have you, **during the last week**, experienced any of the following? | Not  at all | | A bit | | Quite  a bit | | A lot | |
| 42. I have felt older than my own age. | 🞏 | | 🞏 | | 🞏 | | 🞏 | |
| 43. I have felt that I was unlucky. | 🞏 | | 🞏 | | 🞏 | | 🞏 | |
| 44. I have changed diet. | 🞏 | | 🞏 | | 🞏 | | 🞏 | |
| 45. I feel it would have been better not knowing about the aneurism. | 🞏 | | 🞏 | | 🞏 | | 🞏 | |
| 46. I have felt shocked. | 🞏 | | 🞏 | | 🞏 | | 🞏 | |
| 47. I have felt that something is wrong with my body. | 🞏 | | 🞏 | | 🞏 | | 🞏 | |
| 48. I have felt vulnerable at the examination bed. | 🞏 | | 🞏 | | 🞏 | | 🞏 | |
| 49. I have felt that my body was not my own body. | 🞏 | | 🞏 | | 🞏 | | 🞏 | |
| Have you, **during the last week**, experienced any of the following? | Not  at all | A bit | | Quite a bit | | A lot | | Do not know |
| 50. I have felt that the situation seemed hopeless. | 🞏 | 🞏 | | 🞏 | | 🞏 | | 🞏 |
| 51. I have felt sick. | 🞏 | 🞏 | | 🞏 | | 🞏 | | 🞏 |
| 52. I have been worried when I had to push myself. | 🞏 | 🞏 | | 🞏 | | 🞏 | | 🞏 |
| 53. I do not dare to push myself as usual. | 🞏 | 🞏 | | 🞏 | | 🞏 | | 🞏 |

| Have you, **during the last week**, experienced any of the following? | Not  at all | A bit | Quite a bit | A lot | Do not know |
| --- | --- | --- | --- | --- | --- |
| 54. I have felt powerless. | 🞏 | 🞏 | 🞏 | 🞏 | 🞏 |
| 55. I have wondered if I should have taken better care of myself | 🞏 | 🞏 | 🞏 | 🞏 | 🞏 |
| 56. I have experienced mood swings. | 🞏 | 🞏 | 🞏 | 🞏 | 🞏 |
| 57. I have felt that I am getting older. | 🞏 | 🞏 | 🞏 | 🞏 | 🞏 |

| Have you, **during the last week**, experienced any of the following? | Not  at all | A bit | Quite a bit | A lot | Do not know |
| --- | --- | --- | --- | --- | --- |
| 58. I have noted if I could feel something was different in my stomach | 🞏 | 🞏 | 🞏 | 🞏 | 🞏 |
| 59. I have been more tired than usual. | 🞏 | 🞏 | 🞏 | 🞏 | 🞏 |
| 60. I have experienced mood swings. | 🞏 | 🞏 | 🞏 | 🞏 | 🞏 |
| 61. I have felt as though my body was a machine that does not work. | 🞏 | 🞏 | 🞏 | 🞏 | 🞏 |

| Have you, **during the last week**, experienced any of the following? | Not  at all | A bit | Quite a bit | A lot | Do not know |
| --- | --- | --- | --- | --- | --- |
| 62. Less interest in sex. | 🞏 | 🞏 | 🞏 | 🞏 | 🞏 |
| 63. Negative impact on my sex life. | 🞏 | 🞏 | 🞏 | 🞏 | 🞏 |
| 64. Worries about rupture of the aneurism during sexual activities. | 🞏 | 🞏 | 🞏 | 🞏 | 🞏 |

| Have you, **during the last week**, experienced any of the following? | Not  at all | A bit | Quite a bit | A lot | Do not know |
| --- | --- | --- | --- | --- | --- |
| 65. I have missed information about how physical activities can affect my aneurism. | 🞏 | 🞏 | 🞏 | 🞏 | 🞏 |
| 66. I have searched the Internet for knowledge about aneurism rupture. | 🞏 | 🞏 | 🞏 | 🞏 | 🞏 |
| 67. I have looked for knowledge about how the aorta can change. | 🞏 | 🞏 | 🞏 | 🞏 | 🞏 |

| Have you, **during the last week**, experienced any of the following? | Not  at all | | A bit | | Quite a bit | | A lot | | Do not know | |
| --- | --- | --- | --- | --- | --- | --- | --- | --- | --- | --- |
| 68. I have regretted more than usual having smoked all these years. | 🞏 | | 🞏 | | 🞏 | | 🞏 | | 🞏 | |
| 69. I have blamed myself more than usual for having smoked all these years. | 🞏 | | 🞏 | | 🞏 | | 🞏 | | 🞏 | |
| 70. I have felt more than usual that others have blamed me for having smoked all these years. | 🞏 | | 🞏 | | 🞏 | | 🞏 | | 🞏 | |
| 71. I have felt more than usual that others have pointed their finger at me for having smoked all these years. | 🞏 | | 🞏 | | 🞏 | | 🞏 | | 🞏 | |
| 72. I have felt guilty more than usual for having smoked all these years. | 🞏 | 🞏 | | 🞏 | | 🞏 | | 🞏 | |  |
| 73. I have been criticised more than usual by other people for having smoked all these years. | 🞏 | 🞏 | | 🞏 | | 🞏 | | 🞏 | |  |
| 74. I have been angry with myself more than usual for having smoked all these years. | 🞏 | 🞏 | | 🞏 | | 🞏 | | 🞏 | |  |

| If you smoke please answer the following questions: | | |
| --- | --- | --- |
|  | Yes | No |
| 75. After the examination, I have thought about quitting smoking. | 🞏 | 🞏 |
| 76. After the examination, I feel guilty for smoking. | 🞏 | 🞏 |
| 77. After the examination, I feel annoyed with myself for smoking. | 🞏 | 🞏 |
| 78. After the examination, I feel disappointed in myself for smoking. | 🞏 | 🞏 |
| 79. After the examination, I have changed my attitude towards smoking. | 🞏 | 🞏 |
| 80. After the examination, I have had second thoughts about smoking. | 🞏 | 🞏 |

**COS-AAA Part 2:**

| Taking everything into account, has your experience of the AAA Screening Programme caused any of the following:  **Please tick only one box for each question** | | | | | | | | |
| --- | --- | --- | --- | --- | --- | --- | --- | --- |
| 1. After the examinations I have thought about the broader aspects of life: | | | | | | | | |
| 🞏 | | | 🞏 | | 🞏 | 🞏 | 🞏 | |
| Much less | | | Less | | **The same as before** | More | Much more | |
| **Fewer thoughts about life** | | | | |  | **More thoughts about life** | | |
| 2. After the examinations my enjoyment of life is: | | | | | | | | |
| 🞏 | | | | 🞏 | 🞏 | 🞏 | | 🞏 |
| Much less | | | | Less | **The same as before** | More | | Much more |
| **Less enjoyment** | | | | |  | **More enjoyment** | | |
| 3. After the examinations I have been able to relax: | | | | | | | | |
| 🞏 | | | | 🞏 | 🞏 | 🞏 | | 🞏 |
| Much greater | | | | Greater | **The same as before** | Less | | Much less |
| **Worse** | | | | |  | **Better** | | |
| 4. After the examinations my relationship with my family is: | | | | | | | | |
| 🞏 | | | | 🞏 | 🞏 | 🞏 | | 🞏 |
| Much less close | | | | Less close | **The same as before** | Closer | | Much closer |
| **Less close** | | | | |  | **Closer** | | |
|  | | | | | | | | |
| 5. After the examinations my relationship with friends is: | | | | | | | | |
| 🞏 | 🞏 | | | | 🞏 | 🞏 | | 🞏 |
| Much less close | | Less close | | | **The same as before** | Closer | | Much closer |
| **Less close** | | | | |  | **Closer** | | |
| 1. After the examinations my relationship with other people is: | | | | | | | | |
| 🞏 | | | | 🞏 | 🞏 | 🞏 | | 🞏 |
| Much worse | | | | Worse | **The same as before** | Better | | Much better |
| **Worse** | | | | |  | **Better** | | |
| 1. After the examinations my sense of well-being is: | | | | | | | | |
| 🞏 | | | | 🞏 | 🞏 | 🞏 | | 🞏 |
| Much less | | | | Less | **The same as before** | Greater | | Much greater |
| **Less sense of well-being** | | | | |  | **Greater sense of well-being** | | |

| 8. After the examinations my thoughts about the future have been: | | | | |
| --- | --- | --- | --- | --- |
| 🞏 | 🞏 | 🞏 | 🞏 | 🞏 |
| Much less positive | Less positive | **The same as before** | More positive | Much positive |
| **Less positive future** | |  | **More positive future** | |
| 9. After the examinations my sense of well-being is: | | | | |
| 🞏 | 🞏 | 🞏 | 🞏 | 🞏 |
| Much less | Less | **The same as before** | Greater | Much greater |
| **Less sense of well-being** | |  | **Greater sense of well-being** | |
| 10. After the examinations my awareness of life is: | | | | |
| 🞏 | 🞏 | 🞏 | 🞏 | 🞏 |
| Much less | Less | **The same as before** | More | Much more |
| **Less awareness of life** | |  | **Greater awareness of life** | |

| 11. After the examinations I value life: | | | | |
| --- | --- | --- | --- | --- |
| 🞏 | 🞏 | 🞏 | 🞏 | 🞏 |
| Much less | Less | **The same as before** | More | Much more |
| **Value life less** | |  | **Value life more** | |

| 12. After the examinations my energy level is: | | | | | | | | | |
| --- | --- | --- | --- | --- | --- | --- | --- | --- | --- |
| 🞏 | 🞏 | | | 🞏 | | 🞏 | | 🞏 | |
| Much less | Less | | | **The same as before** | | Greater | | Much greater | |
| **Less energy** | | | |  | | **More energy** | | | |
| 13. After the examinations my sense of responsibility for my family is: | | | | | | | | | |
| 🞏 | 🞏 | | | 🞏 | 🞏 | | | 🞏 | |
| Much less | Less | | **The same as before** | | More | | | Much more | |
| **Less responsibility** | | | |  | **More responsibility** | | | | |
| 14. After the examinations I have lived my life to the full: | | | | | | | | | |
| 🞏 | 🞏 | 🞏 | | | | | 🞏 | | 🞏 |
| Much less | Less | **The same as before** | | | | | More | | Much more |
| **Lived life less** | |  | | | | | **Lived life more** | | |
| 15. After the examinations I feel: | | | | | | | | | |
| 🞏 | 🞏 | 🞏 | | | | | 🞏 | | 🞏 |
| Much less  relieved | Less relieved | **As relieved**  **as before** | | | | | More relieved | | Much more relieved |
| **Less relieved** | |  | | | | | **More relieved** | | |
| 16. After the examinations I understand other people’s problems: | | | | | | | | | |
| 🞏 | 🞏 | | | 🞏 | | 🞏 | | | 🞏 |
| Much less | Less | **The same as before** | | | | | Better | | Much better |
| **Less understanding** | | | |  | | **Better understanding** | | | |

| 17. After the examinations I am: | | | | | | |
| --- | --- | --- | --- | --- | --- | --- |
| 🞏 | 🞏 | 🞏 | | | 🞏 | 🞏 |
| Much less  impulsive | Less impulsive | **The same as before** | | | More impulsive | Much more  impulsive |
| **Less impulsive** | |  | | | **More impulsive** | |
| 18. After the examinations my ability to listen to other people’s problems is: | | | | | | |
| 🞏 | 🞏 | | 🞏 | 🞏 | | 🞏 |
| Much less | Less | | **The same as before** | Greater | | Much greater |
| **Less ability to listen** | | |  | **Greater ability to listen** | | |
| 19. After the examinations my desire to try new and unfamiliar things is: | | | | | | |
| 🞏 | 🞏 | | 🞏 | 🞏 | | 🞏 |
| Much less | Less | | **The same as before** | Greater | | Much greater |
| **Less desire** | | |  | **Greater desire** | | |
| 20. After the examinations my desire to try risky things is: | | | | | | |
| 🞏 | 🞏 | | 🞏 | 🞏 | | 🞏 |
| Much less | Less | | **The same as before** | Greater | | Much greater |
| **Less desire** | | |  | **Greater desire** | | |
| 21. After the examinations I have done things that have exceeded my own boundaries: | | | | | | |
| 🞏 | 🞏 | | 🞏 | 🞏 | | 🞏 |
| Much less | Less | | **The same as before** | More | | Much more |
| **Less** | | |  | **More** | | |

**Thank you for your help!**
